# Classifying early infant feeding status from clinical notes using natural language processing and machine learning

**DOI:** 10.1038/s41598-024-58299-x

**Published:** 2024-04-03

**Authors:** Dominick J. Lemas, Xinsong Du, Masoud Rouhizadeh, Braeden Lewis, Simon Frank, Lauren Wright, Alex Spirache, Lisa Gonzalez, Ryan Cheves, Marina Magalhães, Ruben Zapata, Rahul Reddy, Ke Xu, Leslie Parker, Chris Harle, Bridget Young, Adetola Louis-Jaques, Bouri Zhang, Lindsay Thompson, William R. Hogan, François Modave

**Affiliations:** 1 Department of Health Outcomes and Biomedical Informatics, University of Florida College of Medicine, 2004 Mowry Road, Clinical and Translational Research Building, Gainesville, FL 32610 USA; 2 Department of Obstetrics and Gynecology, University of Florida College of Medicine, Gainesville, FL 32610 USA; 3 Division of General Internal Medicine, Department of Medicine, Brigham and Women’s Hospital, Boston, MA 02115 USA; 4 Department of Medicine, Harvard Medical School, Boston, MA 02115 USA; 5 Department of Pharmaceutical Outcomes and Policy, University of Florida College of Medicine, Gainesville, FL 32610 USA; 6 Biomedical Informatics and Data Science Section, Division of General Internal Medicine, Johns Hopkins University School of Medicine, Baltimore, MD 21205 USA; 7 Division of Neonatal and Developmental Medicine, Department of Pediatrics, Stanford University School of Medicine, Palo Alto, CA 94305 USA; 8 Department of Computer and Information Science, Herbert Wertheim College of Engineering, University of Florida, Gainesville, FL 32611 USA; 9 Department of Biobehavioral Nursing Science, University of Florida College of Nursing, Gainesville, FL 32603 USA; 10 Health Policy and Management Department, Richard M. Fairbanks School of Public Health, Indiana University-Purdue University Indianapolis, Indianapolis, IN 46202 USA; 11 Division of Breastfeeding and Lactation Medicine, University of Rochester Medical Center, Rochester, NY 14642 USA; 12 Health Science Center Libraries, University of Florida, Gainesville, FL 32610 USA; 13 Department of Pediatrics, Wake Forest School of Medicine, Winston-Salem, NC 27101 USA; 14 Data Science Institute, Medical College of Wisconsin, Milwaukee, WI 53226 USA; 15 Department of Anesthesiology, University of Florida College of Medicine, Gainesville, FL 32610 USA

**Keywords:** Computational biology and bioinformatics, Computational models, Machine learning

## Abstract

The objective of this study is to develop and evaluate natural language processing (NLP) and machine learning models to predict infant feeding status from clinical notes in the Epic electronic health records system. The primary outcome was the classification of infant feeding status from clinical notes using Medical Subject Headings (MeSH) terms. Annotation of notes was completed using TeamTat to uniquely classify clinical notes according to infant feeding status. We trained 6 machine learning models to classify infant feeding status: logistic regression, random forest, XGBoost gradient descent, k-nearest neighbors, and support-vector classifier. Model comparison was evaluated based on overall accuracy, precision, recall, and F1 score. Our modeling corpus included an even number of clinical notes that was a balanced sample across each class. We manually reviewed 999 notes that represented 746 mother-infant dyads with a mean gestational age of 38.9 weeks and a mean maternal age of 26.6 years. The most frequent feeding status classification present for this study was exclusive breastfeeding [n = 183 (18.3%)], followed by exclusive formula bottle feeding [n = 146 (14.6%)], and exclusive feeding of expressed mother’s milk [n = 102 (10.2%)], with mixed feeding being the least frequent [n = 23 (2.3%)]. Our final analysis evaluated the classification of clinical notes as breast, formula/bottle, and missing. The machine learning models were trained on these three classes after performing balancing and down sampling. The XGBoost model outperformed all others by achieving an accuracy of 90.1%, a macro-averaged precision of 90.3%, a macro-averaged recall of 90.1%, and a macro-averaged F1 score of 90.1%. Our results demonstrate that natural language processing can be applied to clinical notes stored in the electronic health records to classify infant feeding status. Early identification of breastfeeding status using NLP on unstructured electronic health records data can be used to inform precision public health interventions focused on improving lactation support for postpartum patients.

## Introduction

Human milk is the optimal source of nutrition for infant health and development^1,2^. The benefits of human milk include greater neurocognitive development and protection against infection, gastroenteritis, respiratory infections, obesity, diabetes, childhood leukemia, and sudden infant death syndrome^1,2^. The World Health Organization advises that infants should be exclusively breastfed for the initial 6 months of their lives and continue to receive breast milk alongside suitable solid foods until the age of two years or more^3^. Despite this opportunity in preventative health, recent statistics from the Centers for Disease Control and Prevention (CDC) show that although approximately 8 out of 10 (83.9%) infants in the U.S. start breastfeeding at birth, merely 1 out of 4 (26.8%) continue to breastfeed exclusively for 6 months^4^. Further, in-hospital formula feeding during the first 48 h of life is associated with a shorter duration of exclusive breastfeeding, and supporting lactation during the early postpartum period is critical for optimizing long-term milk volume^5–8^. Early identification of in-hospital breastfeeding status presents an important opportunity for directing population-level interventions focused on improving exclusive breastfeeding rates.

Electronic health records (EHRs) represent a longitudinal repository of structured and unstructured data that can enable large-scale evaluation of health outcomes that breastfeeding outcomes^9,10^. Despite the relative accessibility of structured EHR data, large-scale investigation of breastfeeding is hindered by missing data^11^, lack of standardized templates^12^, and national registries that require manual review by frontline health care workers^12^. In contrast, clinical notes are comprised of unstructured narratives and other free text written by clinicians that contain rich subjective data. Due to the limitations of manual chart review, the examination of clinical notes requires the use of natural language processing (NLP)^10^. Natural language processing is a field of research that has produced methods intended to extract meaning (“semantics”) from natural language text, such as the unstructured data found in clinical notes^13^. Given the rate at which unstructured clinical information is created, automated solutions such as NLP are vital for analyzing texts efficiently and extracting useful infant feeding data^10^.

Natural language processing has been successful in examining clinical notes to identify complex maternal and pediatric conditions including severe maternal morbidity^14^, child respiratory illness^15^, neurodevelopment after preterm birth^16^, postpartum self-harm^17^, and infant feeding in the presence of congenital heart defects^18^. More recently, NLP methods focused on breastfeeding were used to extract lactation-specific drug information from US Food and Drug Administration–approved medication labels and the publicly available National Library of Medicine Drugs and Lactation Database^19^. However, the use of NLP to analyze clinical notes to identify breastfeeding status in early postnatal populations has yet to be explored. This study leverages NLP methods to classify clinical notes according to infant feeding status using de-identified linked maternal-infant EHRs collected from the University of Florida (UF) Health Integrated Data Repository (IDR), a large-scale clinical data warehouse that securely stores US Health Insurance Portability and Accountability Act (HIPAA)-compliant and institutional review board-approved data sets^20^.

## Methods

### Data source and study population

Electronic health records for mothers and their infants, which had been de-identified and linked, were gathered from UF Health Shands Hospital in Gainesville, Florida, United States between June 1, 2011, and April 30, 2017. UF Health is a teaching hospital and major referral center serving a largely rural population in north central Florida and neighboring regions. The obstetric unit is a 27-bed, Baby-Friendly accredited facility. A trustworthy intermediary from the UF Health IDR utilized fully identified data to execute the linkage, subsequently stripping away identifiers from the connected dataset prior to distributing the de-identified data to the research team^21^. Demographic details, including the type of insurance at the time of delivery, age, race, and ethnicity, were obtained from the maternal records, while information on each infant's sex, race, and ethnicity was derived from the infant's records. It is important to note that, as part of standard care, all infant records within the UF Health system are connected to their respective maternal records. Our study included infants born within the UF Health system who had attended at least one postnatal well visit. Given that infant feeding information is frequently collected across multiple clinical settings, we included all note types in our corpus development. We included all clinical notes collected within the first 24 h of delivery and included ambulatory notes, discharge summaries, emergency department (ED) observation progress notes, labor and delivery notes, lactation notes, progress notes, significant event notes, and operative notes, as well as pathology, cardiology, endoscopy, pulmonary, and radiology reports. We developed our corpus by reading through 30 to 50 random notes to develop a library of keywords related to infant feeding (Supplementary Information). We then evaluated each clinical note for the presence of at least one keyword from our library. The analysis corpus was created by randomly selecting notes from the eligible cohort and implementing a rule whereby 80% of the notes had to have at least one keyword. As illustrated by Fig. S2, our inclusion criteria for this study included all patients with at least one postnatal visit, a delivery visit, and at least one clinical note within ± 2 days of the baby's date of birth. We further enriched our corpus through random sampling to include keywords with the following proportions: 40% breastfeeding, 40% express/pump feeding, and 20% non-breast and pump feeding. The final analysis corpus included 999 clinical notes that were linked to 746 patients. The research adhered to the principles of the Declaration of Helsinki and received approval from the University of Florida's Institutional Review Board (IRB201601899). Informed consent was waived by the Institutional Review Board of the University of Florida. The original dataset was made anonymous in alignment with HIPAA regulations^22^.

### Chart review to obtain gold standard labels

Our analysis corpus included medical record reviews of 999 clinical notes performed by two trained annotators. Clinical notes were classified according to infant feeding status using Medical Subject Headings (MeSH) terms. In this analysis, infant feeding status was classified as (1) exclusive breastfeeding (D001942), (2) exclusive formula/bottle feeding (D001903), and Not-Related. Infants who were fed expressed mother’s milk (using a breast pump) were classified as breastfeeding. We only focused on the quantitative infant feeding information pertaining to a specific patient and excluded general or instructional information such as the breastfeeding information mentioned in guidelines (e.g., “we discussed guidelines with the patient on breastfeeding”). All clinical notes were annotated using TeamTat, a clinical language annotation tool that includes corpus quality assessment via inter-annotator agreement statistics and an interface for annotation review and inter-annotator disagreement resolution^23^. Two reviewers examined each chart, and any discrepancies were settled by a third reviewer to reach agreement on the infant feeding status. This process also involved resolving any disagreements that arose between the initial two reviewers. The conflicts that typically resulted between annotators generally were either syntactic or semantic conflicts. Syntactic conflicts included situations when one annotator would highlight a whole sentence compared to another annotator highlighting a phrase within that sentence. Semantic conflicts included annotators deciding on whether or not a particular manner of wording within a note constituted or implied a particular form of infant feeding status. After resolving conflicts through discussion, we updated the annotation guide to mitigate the occurrence of similar future conflicts, increasing annotator agreement rates over the course of each project. We had substantial agreement among annotators, with an average inter-annotator agreement of 0.62 that ranged from 0.6 to 0.7 prior to resolving conflicts as a group. We have included an expanded discussion of infant feeding classes (Table S2) and the corpus annotation strategy as Supplementary Information.

### Natural language data processing

Our NLP workflow included the use of the TeamTat tool for clinical language annotation and Python^24^ scripts for preprocessing of clinical notes and the modeling for NLP analysis. Preprocessing of note text included converting all words to lowercase and removing punctuation, numerical values, and stop words. After preprocessing the notes, the remaining words were lemmatized to their base form using the WordNet Lemmatizer. To address class imbalance, downsampling was performed to create a test corpus to ensure an equal number of samples in each class. The downsampling specifically targeted the NA class, which exhibited an imbalance compared to other classes. Prior to downsampling, the dataset had imbalance ratio of 3.428 between the majority (NA) and minority classes (bottle feeding). The dataset is split into training and testing sets using a 70:30 ratio. The term frequency-inverse document frequency (TF-IDF) vectorizer is employed to convert the text data into numerical features. The training data are used to fit the vectorizer, and both the training and testing data are transformed into TF-IDF representations. Next, the class labels are encoded into numeric values using LabelEncoder. This allows the XGBoost classifier to work with the encoded labels. The XGBoost classifier is then trained using the training data and hyperparameter tuning with GridSearchCV. The best model and its corresponding hyperparameters are identified based on cross-validation performance. Using the best model, predictions are made on the testing data. The predicted class labels are inverse transformed to obtain the original class labels. Various evaluation metrics such as accuracy, precision, recall, and F1 score are calculated to assess the model's performance. Table S3 outlines the distribution of feeding classes that were used for the analysis, training, and test data.

### Machine learning models

We implemented supervised learning models for the classification task. Python^24^ and scikit-learn package^25^ were used for the model development. We did not use any dimension reduction techniques and feature extraction included all note words that passed preprocessing. The machine learning models chosen for comparison include logistic regression^26^, XGBoost^27^, support vector classifier, random forest, and k-nearest neighbors^28^. Specifically, logistic regression is a linear model that converts linear regression with sigmoid function, making it a better fit for two-classification problems. It has been widely used for health-related problems and has achieved promising performances^29–31^. The “one-vs-the-rest” algorithm, also known as “one-vs-all,” is a common approach used by logistic regression to handle multiclass classification. Similarly, support vector classifiers (SVCs) also employ the one-vs-the-rest strategy for multiclass classification tasks. The k-nearest neighbors (KNN) algorithm categorized each sample in the testing data by looking at the “k” samples in the training data that were closest to the test sample. Then, it identified the most common class (category) among these “k” nearest samples. This common class was then assigned to the test sample. All models were trained with 70% training data and evaluated on 30% test data. Our balanced dataset was completed at the note level and included 507 notes that were split into test (n = 355 notes) and training (n = 152 notes) sets. Hyperparameters were optimized with fivefold cross-validations using the training data.

### Ethic statement

The study was conducted in accordance with the Declaration of Helsinki and approved by the Institutional Review Board of the University of Florida (IRB201601899).

### Informed consent statement

Informed consent was waived by the Institutional Review Board of the University of Florida.

## Results

Table 1 presents the characteristics of patients who delivered an infant at UF Health Shands Hospital between June 2011 to April 2017. Patient demographics within the analysis cohort were not significantly different from the full or eligible cohort across perinatal variables, including maternal age, gestational age of the infant, and birth weight. Supplementary Table S1 includes a complete description of the corpus. Briefly, each note in the analysis corpus contained a mean of 776.6 characters, 115.7 words, and 9.3 sentences. Table 2 outlines the classification of clinical notes in the analysis cohort according to infant feeding status. The most frequent feeding status classification present for this study was breastfeeding [n = 183 (18.3%)], then formula/bottle feeding [n = 146 (14.6%)], followed by pump/expressed feeding [n = 102 (10.2%)], with mixed feeding being the least frequent [n = 23 (2.3%)]. After merging the breastfeeding and express/pump classes, Table S3 shows that nearly 1 in 3 notes (28.5%) were classified as breastfeeding. Using unbalanced data (Table S4), our averaged performance across all models was 72.4% and the best performing models were SVC (81.1%) and XGBoost (80.1%). After balancing the data (Table 3**)**, the averaged performance across all models was 76.6%**.** Within the balanced data, our machine learning results revealed the XGBoost model outperformed all others by achieving an accuracy of 90.1%, a macro average precision of 90.3%, a macro average recall of 90.1%, and a macro average F1 score of 90.1%. By majority vote, we also implemented an ensemble method based on algorithms used in this study and found the F1 score was generally consistent with our results (data not shown).

**Table 1:** Patient characteristics for training and testing sets.

Variables	Entire cohort	Eligible cohort	Analysis cohort
N	16,108	1888	746
Note number	659,576	55,699	999
Maternal age (yrs)	27.6 ± 6.0	27.3 ± 5.8	26.6 ± 5.9
Gestational age (wks)	38.2 ± 3.2	38.9 ± 2.0	38.9 ± 2.1
Birth weight (g)	3075.9 ± 738.4	3179.8 ± 565.9	3144.4 ± 569.0
Gender			
Female	7799 (48%)	940 (50%)	369 (49%)
Male	8308 (52%)	948 (50%)	377 (51%)
Infant feeding present (%)	12.49	16.31	80.2

**Table 2:** Distribution of infant feeding classes in the unbalanced analysis corpus.

Classes	Analysis (n = 999) (%)
Breastfeeding	183 (18.3)
Bottle feeding	146 (14.6)
Mixed feed	23 (2.3)
Pump/express	102 (10.2)
NA: feeding related	127 (12.7)
NA: not related	418 (41.8)

**Table 3:** Model comparison using balanced data.

Model	Accuracy	Precision	Recall	F1 Score
SVC	0.738	0.739	0.738	0.737
Logistic regression	0.745	0.744	0.745	0.743
XGB classifier	**0.901**	**0.903**	**0.901**	**0.901**
Random forest classifier	0.843	0.842	0.843	0.842
K neighbors classifier	0.601	0.593	0.601	0.583

Estimates are averages across all models and the best performing model is identified in bold text.

## Discussion

### Overview

In-hospital infant feeding patterns established during the first 48 h of life have been significantly associated with the duration of excusive breastfeeding^32^. This study found that applying a validated NLP-based workflow and machine learning to clinical notes was a technically feasible and highly accurate method of estimating in-hospital infant feeding patterns during the first 24 h postnatal. The overarching goal for this analysis was to develop simple infant feeding classification methods using machine learning that can be easily implemented within pediatric health care centers. Our results revealed only a modest difference in the averaged performance across all models tested using balanced and unbalanced data (~ 4%). To our knowledge, this is the first NLP-based cross-sectional study that identifies perinatal health behavior such as breastfeeding status from clinical notes. Our results highlight the translational potential for NLP-based tools to facilitate early identification of breastfeeding status in postpartum patients. The primary contribution of this study is twofold:

#### Breastfeeding outcomes

First, we have developed an NLP-based method to extract infant feeding status from unstructured EHR data that can be used to enhance population estimates of breastfeeding. Previous estimates of population-level breastfeeding have traditionally relied on self-reporting^33^, phone call screenings^34^, and mobile apps or registries that are costly^34^, labor intensive, and may be subject to selection bias^35^. The adoption of EHRs has enabled structured data analysis to estimate population-based breastfeeding outcomes in Canada^33,36^ and Europe^37^ using regression^37^ and rule-based methods. The limitations of structured EHRs to evaluate breastfeeding include low data quality^12^, high levels of missing data^38^, EHRs-system constraints and the absence of standardized breastfeeding-related definitions and external mandates for structured data. An important observation from our study is that NLP is a feasible method to extract perinatal outcomes such as infant feeding status from unstructured EHR data that include clinical notes, pathology and radiology reports, and continuity of care documents^39–41^. Our study extends the area of perinatal NLP to include classification of infant feeding status using standard biomedical ontologies (e.g., MeSH) and rigorous annotation guidelines that can be used to standardize breastfeeding outcomes extracted from unstructured EHR data.

#### Social and behavioral determinants of health

The secondary contribution of our work is the development of multilevel tools to extract data on infant feeding as a behavioral determinant of infant and mother health. Social and behavioral determinants of health (SBDH) are powerful drivers of morbidity, mortality, and well-being, and may contribute to perinatal outcomes^42,43^. Previous work on utilizing NLP for identifying SBDH has focused on factors such as substance abuse, alcohol and tobacco use, housing, and food insecurity^44^. However, existing SBDH and health behaviors (e.g., smoking, alcohol, substance abuse) have not considered perinatal populations. Our developed methods can be extended to identify relevant SBDH in perinatal populations.

### Strengths

We show multiple classification methods of training clinical notes to identify infant feeding types. The fact that simpler models (logistic regression, decision tree, and SVC) perform better indicates that the current infant feeding types are fairly distinguishable given the size and number of features of the annotated corpus. We created a corpus of fully manually reviewed clinical data (999 notes), which provides a high-quality training and evaluation resource for other clinical NLP tasks. Additionally, the included clinics in this study serve patients with diverse socioeconomic statuses, suggesting the potential generalizability of our methods. Notably, we have achieved promising results using conventional machine learning algorithms, with the advantages of feasibility and explainability. Using NLP, hospitals can quickly and regularly characterize infant feeding trends in their own populations without waiting for annual surveys to publish breastfeeding trends. Our model achieved its highest accuracy of 90% on the test data when trained on the balanced dataset, emphasizing the significance of addressing class imbalance for improved performance. In contrast, the accuracy with unbalanced data was 80%, highlighting the positive impact of downsampling on the model's ability to generalize across different classes. Additionally, the national survey results frequently do not include underrepresented groups, likely due to selection bias. As a result, the statistics may not be generalizable to certain delivery hospitals. NLP can lay the groundwork for community awareness of current infant feeding practices and thus targeted interventions among all patient groups.

### Limitations

There are limitations to our study, including the fact that we used data from a single health system and the terminology used by providers at other institutions to describe infant feeding may differ from that used in the current study. Given the size of our corpus and distribution of infant feeding classes, we were underpowered to classify breastfeeding and pump/express independently with > 90% accuracy. The classification of infant feeding status used in our final analysis included exclusive breastfeeding, exclusive formula/bottle feeding, and Not-Related. Infants who were fed expressed mother’s milk (using a breast pump) were classified as breastfeeding. The classification of “bottle feeding,” as asked at UF Health upon the pregnant mother’s birth hospital admission, is likely meant as a synonym for “formula feeding intent” but unfortunately is an inaccurate substitute. “Bottle feeding” has dual meaning and thus remains an ambiguous label for infant feeding. Bottle contents can include either breast milk or formula milk. Use of “bottle feeding” as a category can cause imprecise estimates of benchmark outcome measures such as formula supplementation in hospital. For term, healthy infants, such as in our study, bottles may be used to provide expressed breast milk when parents return to work, as a parental preference, or due to infant medical needs. The limitation of using “bottle fed” as a surrogate for “formula” illustrates the need for a consensus definition of infant feeding including categorizing feeds into clinically relevant categories to inform epidemiologic measurements and above programs. Additionally, given that infant feeding information is collected across multiple clinical settings, we included all note types in our corpus development. However, we did not evaluate the influence of note type and/or how selections within a given note influenced model performance. Working with stakeholders, including EHRs companies, to ensure the most accurate input information will result in the maximum potential to utilize NLP for the most meaningful clinical data output.

### Future directions

In addition to developing a consensus definition of early infant feeding, future restructuring of NLP categories can align hospital data with nationally collected data. Conducted by the CDC, the National Immunization Survey–Child focuses on 3 early infant feeding questions as follows: (1) Was [child] ever breastfed or fed breast milk? (2) How old was [child’s name] when [child’s name] completely stopped breastfeeding or being fed breast milk? (3) How old was [child’s name] when (he/she) was first fed formula? Using NLP as a tool to examine linked mother-infant dyad records on birth hospital admission and pediatric outpatient visits has the potential to define breastfeeding rates as per the CDC’s classifications of “ever breastfed” or “exclusive breastfeeding,” examining duration time points of 3 months, 6 months, 12 months, 18 months, and in the future even up to 24 months. With the participation of multiple hospitals, NLP may be able to provide local, state, and federal stakeholders with quarterly updates as an alternative to annual update reports from CDC. Clinically, some EHRs can easily generate data reports, such as “intention to feed” data entered in drop-down boxes, with predetermined options including breast, bottle, or both. However, some data only exist in the bodies of certain notes. An important example is status and type of breast pump for the lactating mother. Since this information is only in the body of the lactation consultant’s note, it would not be in an EHR report but instead could be made available by utilization of NLP. If there are social or economic barriers to obtaining a breast pump, insurance, including Medicaid, will cover a breast pump. Using NLP to automatically extract data regarding social or economic barriers to obtaining a breast pump from clinical notes in real time will allow physicians, nurses, or lactation consultants to intervene during a critical window of lactation, which may affect the mother’s potential lactation duration. Furthermore, future experiments with infant feeding classification methods using more advanced data preprocessing approaches like FastText, Word2Vec, Doc2Vec, and BERT, more advanced ensemble-like stacking, open-source large language models like LLaMa^45^, and unsupervised learning (such as clustering and anomaly detection) can be considered. Additionally, adding structured EHR data may further improve the model’s performance^46^, and structured data such as demographics may also impact breastfeeding outcomes^47^.

## Conclusion

In this study, we developed an NLP-based method for breastfeeding outcome identification from clinical notes. We believe that NLP-based identification of infant feeding status has potential as an optimal solution that will increase the accuracy of documentation of infant feeding status and can be used to target interventions to support breastfeeding in at-risk patients. Implementing our approach will minimize the impact of current documentation routines while providing structured data regarding breastfeeding outcomes for clinicians to guide clinical care. In the future, we will integrate structured EHR data to further improve the model’s performance.

### Supplementary Information

Supplementary Information.

## Data Availability

Data is available upon request. Please contact the corresponding author, Dominick Lemas (djlemas@ufl.edu) to request the data.
