# Supplementary material for: Classifying early infant feeding status from clinical notes using natural language processing and machine learning
Source: Sci Rep. 2024 Apr 3;14:7831. doi: 10.1038/s41598-024-58299-x (PMC10991582; doi:10.1038/s41598-024-58299-x)
Supplement: Supplementary file 1 — Supplementary Information. [file 41598_2024_58299_MOESM1_ESM.docx]

**Classifying early infant feeding status from clinical notes using natural language processing and machine learning**

Dominick J. Lemas, PhD^1,2*^, Xinsong Du^3,4^, Masoud Rouhizadeh, PhD^5,6^, Braeden Lewis^1^, Simon Frank^1^, Lauren Wright^1^; Alex Spirache^1^, Lisa Gonzalez^1^, Ryan Cheves^1^, Marina Magalhães^7^; Ruben Zapata^1^, Rahul Reddy^8^; Ke Xu^1^; Leslie Parker, PhD^9^; Chris Harle, PhD^10^; Bridget Young, PhD^11^, Adetola Louis-Jaques, MD^2^; Bouri Zhang, PhD^12^; Lindsay Thompson, MD^13^; William R. Hogan, MD^14^; François Modave, PhD^15^

^1^ Department of Health Outcomes and Biomedical Informatics, University of Florida College of Medicine, Gainesville, Florida 32610, USA

^2^ Department of Obstetrics and Gynecology, University of Florida College of Medicine, Gainesville, Florida 32610, USA

^3^ Division of General Internal Medicine, Department of Medicine, Brigham and Women’s Hospital, Boston, Massachusetts 02115, USA

^4^ Department of Medicine, Harvard Medical School, Boston, Massachusetts 02115, USA

^5^ Department of Pharmaceutical Outcomes and Policy, University of Florida College of Medicine, Gainesville, Florida 32610, USA

^6^ Biomedical Informatics and Data Science Section, Division of General Internal Medicine, Johns Hopkins University School of Medicine, Baltimore, Maryland 21205, USA

^7^ Division of Neonatal and Developmental Medicine, Department of Pediatrics, Stanford University School of Medicine, Palo Alto, California 94305, USA

^8^ Department of Computer and Information Science, Herbert Wertheim College of Engineering, University of Florida, Gainesville, Florida 32611, USA

^9^ Department of Biobehavioral Nursing Science, University of Florida College of Nursing, Gainesville, Florida 32603, USA

^10^ Health Policy and Management Department, Richard M. Fairbanks School of Public Health, Indiana University–Purdue University Indianapolis, Indianapolis, Indiana 46202, USA

^11^ Division of Breastfeeding and Lactation Medicine, University of Rochester Medical Center, Rochester, New York 14642, USA

^12^ Health Science Center Libraries, University of Florida, Gainesville, Florida 32610, USA

^13^ Department of Pediatrics, Wake Forest School of Medicine, North Carolina 27101, USA

^14^ Data Science Institute, Medical College of Wisconsin, Milwaukee, Wisconsin 53226, USA

^15^ Department of Anesthesiology, University of Florida College of Medicine, Gainesville, Florida 32610, USA

* Corresponding author: Dominick J. Lemas, PhD, Assistant Professor, Department of Health Outcomes and Biomedical Informatics, University of Florida College of Medicine, 2004 Mowry Road, Clinical and Translational Research Building, Gainesville, FL 32610; Phone: 352-294-5971; Email: djlemas@ufl.edu

**Annotation Guidelines for Infant Feeding Classification**

Lemas Laboratory- University of Florida

**Introduction:** The type of feeding that a mother provides their infant correlates to different health care outcomes for both mother and baby. A host of socioeconomic factors and personal decisions influence what mothers choose as the type of feeding that they provide to their infant. This annotation guide seeks to create standardized guidelines for annotation classifications and instances of infant feeding. The categories of infant feeding are as follows; breastfeeding, bottle feeding, breast milk expression/breast pump, mixed (both formula and breastmilk), NA but infant feeding related, and NA not infant feeding related.

**Description of Project:** We seek to generate a large corpus that has enough data to accurately reflect the real population of mothers in this country and the different scenarios that influence infant feeding types and health care outcomes. This document will provide guidelines aimed at disambiguating clinical notes that relate to infant feeding. These guidelines will help annotators mark unambiguous instances of infant feeding and classify them into a unique category.

**The Annotation Task:** The annotation in this project is two-fold—we wish to identify both the classification and instances of infant feeding. This information is extracted via clinical notes. Every document will have a classification of the type of feeding found in section 1. For every document with instances of infant feeding, the sentence that contains the instance of feeding will also be marked. Again, by the list provided in section 1. Every sentence annotated should include the period or relevant punctuation.

**Annotation Techniques:** When annotating instances of infant feeding, it is crucial to include all necessary information in the annotation. Full sentences including punctuation is best practice for annotating instances of feeding. This eliminates ambiguity and allows us to extract the best information from each annotation. For instances that are especially long, it is sufficient to include the relevant subject and predicate that contains the feeding instance. Make sure that the relevant data is included in the annotation. You will find that most notes *do not* relate to infant feeding, that is to be expected. Make sure to highlight the NA classification if the note does not pertain to infant feeding *in any way.* If there are no instances, yet infant feeding is implied, select NA-feeding related. Relevant examples can be found below in sections (5) and (7).

**Definitions of the Categories:** This project has two classes that include: FEED_INSTANCE and FEED_CLASS. A FEED_INSTANCE is simply an instance of infant feeding of any classification. The FEED_CLASS is the method of infant feeding, ie. breastfeeding, bottle feeding, etc.

FEED_INSTANCE

- Mother reports that baby just fed on the L breast, which was palpably softer than the right breast. BREAST
- The patient had just Finished feeding the baby off the breast. BREAST
- Mob demonstrated hand expression. EXPRESS/PUMP
- Pt has been formula feeding infant. BOTTLE

These are definitions of the categories established in section 1. Each term is also listed with their unique MESH ID to avoid ambiguity.

- Breastfeeding: The nursing of an infant at the breast. MESH ID: D001942
- Bottle Feeding: Use of nursing bottles for feeding. MESH ID: D001903
- Breast Milk Expression/Breast Pump: The act of evacuating breast milk by hand or with a pump. MESH ID: D061186
- Mixed Feeding: Mixture of feeding types, specifically formula/bottle and breast. MESH ID: MIXED

**Criteria for Annotation:** It is essential that we adhere to the strict clinical guidelines as they relate to infant feeding. This ensures that we are as accurate as possible with all annotations. Below is a list that is meant to serve as a guide for the classification of feeding type.

The BREAST classification will be selected only when breastfeeding is the exclusive method of infant feeding. This is consistent with clinical guidelines. BOTTLE will be selected with instances that are consistent with bottle-feeding. The MIXED classification is appropriate for notes that include both breastfeeding and bottle-feeding. PUMP/EXPRESS will be present tense instances whereby the only other instances will be BREAST, could be past tense. IF both breast and PUMP/EXPRESS are present tense, select PUMP as we can infer those infants were also breastfed, but it cannot be inferred that all breastfed infants fed via pump/express.

**Rules to Reduce Ambiguity and Build Consensus:** Many notes are ambiguous, this makes it difficult to determine if infant feeding is being described, the classification of feeding, the content of the note, etc. To handle this, be sure to pay special attention to the canonical description of feeding within the note. Statements of desire or intent are *not* instances of feeding, we cannot imply that a mother who wishes to breastfeed actually breastfed their infant. Instead, make sure that instances of feeding described in the notes are ongoing, or in the recent past. Do not include future tense, or statements of intent/desire as instances of feeding. Communication with your team is essential for getting the most from every annotation.

A visual representation is provided below in the form a flow chart:

IF the feeding instance is in present tense (ie. mother **is feeding**, Baby **is latched** onto breast) → select the instance for annotation

IF the feeding instance is in past tense → look for the timeframe in which the instance occurred (ie. Mother **just finished** bottle feeding == instance vs. Mother **previously breastfed** her other children != instance) → recent past == instance, remote past != instance

IF the feeding instance is described in future tense OR statements of intent/will are described → do NOT annotate these instances, we cannot infer that feeding occurred. (ie. MOB **wishes** to bottle feed != instance, I educated the patient on the benefits of breastfeeding != instance)

**Annotation of Documents and Examples:** You will notice that for every document, only *one* classification (breastfeeding, bottle, etc.) is chosen. It is crucial that only one label be chosen for each document. While we may have multiple instances of feeding each document can only have one classification. You will also notice that some sentences are ambiguous, meaning that the classification of feeding is not immediately clear. For example, words that convey intent or desire are used, but they do not necessarily mark a canonical instance of feeding. These guidelines aim to assist in the disambiguating of clinical notes.

**Example 1**

Pt plans to do both breastfeeding and formula. Mother reported to had previously breastfed in L/D at 09:30.

REASON: While there is intent to do mixed feeding, this note only describes breastfeeding. Breastfeeding is the sole method described, so BREAST is the classification.

FEED_CLASS: BREAST

**Example 2**

Called Dr. [**NAME**] to room to assess pt bleeding after several large clots expressed.

REASON: This note does not relate to infant feeding in any way.

FEED_CLASS: NA is the correct class.

**Example 3**

Infant Feeding: bottle

REASON: Some notes are very straightforward; the classification is clearly provided. Clearly a bottle classification and instance of bottle feeding.

FEED_CLASS: BOTTLE is the correct class.

**Example 4**

Pt states breastfeeding is going well

REASON: Present progressive tense (‘is going’) signifies that breastfeeding is

on-going. Classification of breastfeeding and an instance of breastfeeding.

FEED_CLASS: BREAST is the correct class.

**Example 5**

Report from ** that pt is pumping, and feeding formula per her choice.

REASON: Here we can see the classification of mixed feeding. The presence of both formula and breastmilk (pumping here) is mixed feeding. Both feeding types are instances of feeding too.

FEED_CLASS: MIXED is the correct class.

**Example 6**

Lactation Note: Grandma was giving the baby formula during rounds. Mom states that she breast fed first then the formula was given.

REASON: We can see another classification of mixed feeding here. The feeding of both breastmilk and formula is infant feeding. We also have one instance of bottle feeding and one instance of breastfeeding.

FEED_CLASS: MIXED is the correct class.

**Example 7**

Pt has started breast pumping. Reviewed care plan including getting up with assistance, breast pumping and pain management.

REASON: Unambiguously breast pump classification with an instance of breast pump as feeding.

FEED_CLASS: EXPRESS/PUMP is the correct class.

**Example 8**

Mob with tender nipples. Lactation called and made aware. Pt told to call when infant is nursing. Pt has lanolin. Mob aware on how to do skin to skin and hand expression.

REASON: While this note does not contain any information to classify the type of feeding or instances of infant feeding, it is clearly still *related* to infant feeding. So, this would be classified as NA- feeding related.

FEED_CLASS: NA-FEEDING RELATED is the correct class

**Example 9**

She was able to use both the football and *********** positions with ease and comfort. She verbalized satisfaction with her nursing session.

REASON: While breastfeeding is not explicitly mentioned here, based on the context and other words used we can see that this is an instance of breastfeeding. Terms like ‘football hold’ and ‘nursing’ inform our decision here.

FEED_CLASS: BREAST is the correct class

**Example 10**

Patient medicated for uterine cramping with motrin 600 mg po

REASON: A large proportion of notes are unrelated to infant feeding. This is another case of that. Here, we would use the classification NA- not infant feeding related.

FEED_CLASS: NA

**Supplementary Tables**

**Table S1** **Corpus characteristics**

|  | **Entire Cohort** | **Eligible Cohort** | **Analysis Cohort** |
| --- | --- | --- | --- |
| Patients | 16108 | 1888 | 746 |
| Notes | 659576.0 | 55699.0 | 1000.0 |
| Mean Sentence Count | 6.04 | 5.99 | 9.32 |
| STD Sentence Count | 7.77 | 7.25 | 8.95 |
| Mean Word Count | 57.82 | 58.38 | 115.67 |
| STD Word Count | 100.66 | 95.55 | 127.08 |
| Mean Character Count | 377.16 | 386.14 | 776.56 |
| STD Character Count | 670.83 | 644.42 | 850.26 |
| % Notes Related to Feeding | 12.49 | 16.31 | 80.2 |

**Classification Appendix**

**Table S2** **Descriptions of infant feeding classes**

| Classification Label | MeSH ID | Definition | Example |
| --- | --- | --- | --- |
| BREAST | D001942 | The nursing of an infant at the breast. | Mother reports that baby just fed on the L breast, which was palpably softer than the right breast. |
| EXPRESS/PUMP | D061186 | The act of evacuating breast milk by hand or with a pump. | Mob demonstrated hand expression |
| BOTTLE | D001903 | Use of nursing bottles for feeding. | Pt has been formula feeding infant. |
| MIXED | MIXED | Mixture of feeding types, specifically formula/bottle and breast. | Report from ** that pt is pumping and feeding formula per her choice. |
| NA: NOT RELATED | NA: NOT RELATED | Not related to infant feeding in any discernible way. | Called Dr. [**NAME**] to room to assess pt bleeding after several large clots expressed. |
| NA: RELATED | NA: RELATED | Infant feeding implied, but not explicit. | Mob with tender nipples. Lactation called and made aware. Pt told to call when infant is nursing. Pt has lanolin. Mob aware on how to do skin to skin and hand expression. |

**Table S3 Class distribution for balanced corpus across analysis, training and test data**

| **Classes** | **Analysis (n = 999)** | **Balanced (n = 507)** | **Training (n = 355)** | **Test (n = 152)** |
| --- | --- | --- | --- | --- |
| Breastfeeding | 285 (28.5%) | 169 (33.3%) | 118 | 51 |
| Bottle Feeding | 169 (16.9%) | 169 (33.3%) | 118 | 51 |
| Mixed Feed | 545 (54.6%) | 169 (33.3%) | 119 | 50 |

**Table S4** **Model comparison using unbalanced data**

| **Model** | **Accuracy** | **Precision** | **Recall** | **F1 Score** |
| --- | --- | --- | --- | --- |
| SVC | 0.811 | 0.767 | 0.681 | 0.697 |
| Logistic Regression | 0.784 | 0.811 | 0.588 | 0.601 |
| XGBoost | 0.801 | 0.766 | 0.700 | 0.719 |
| Random Forest | 0.553 | 0.263 | 0.254 | 0.188 |
| K Neighbors Classifier | 0.674 | 0.504 | 0.435 | 0.415 |

**Supplementary Figures**


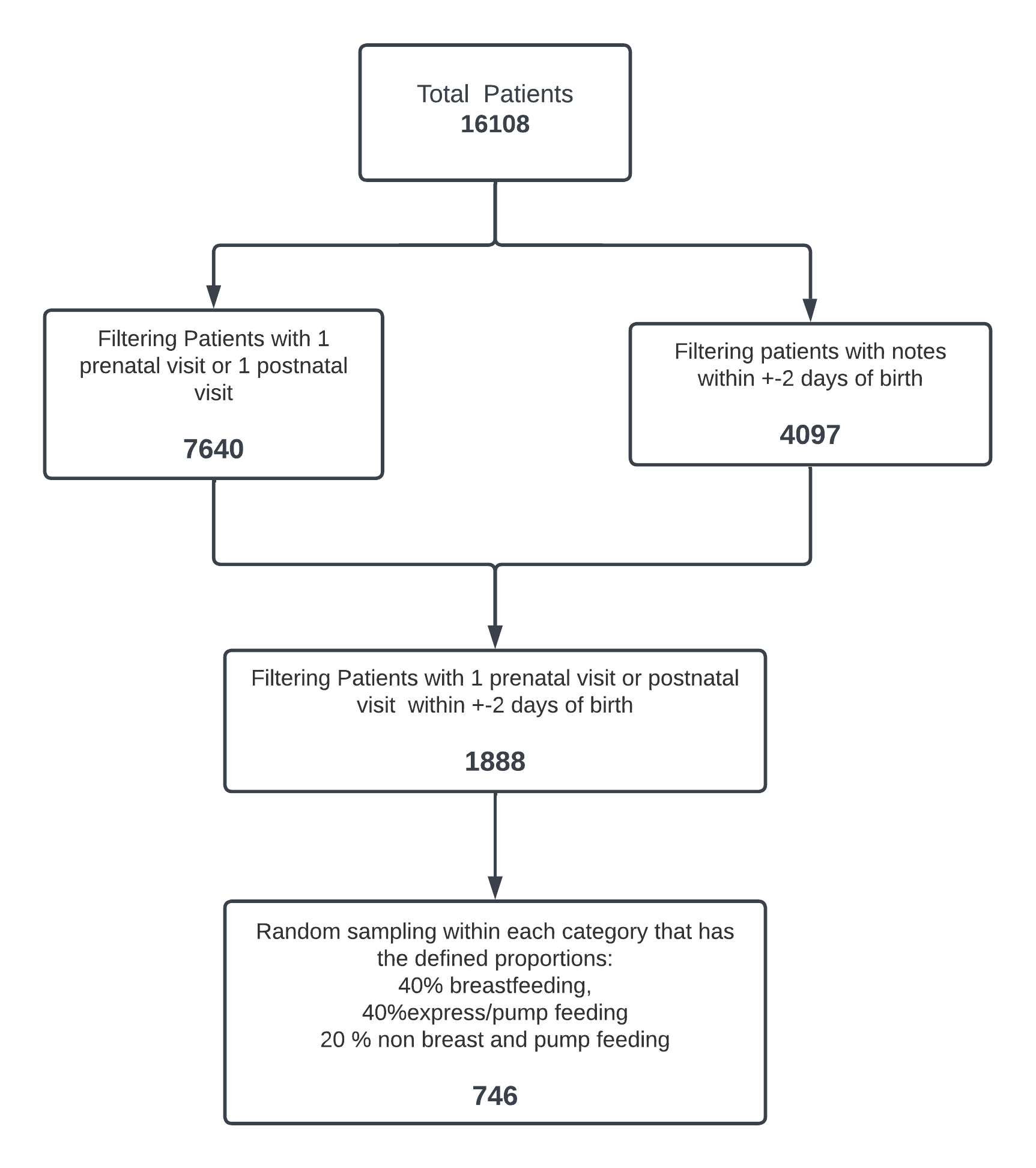


**Figure S1**. Flow chart of patients from UF Health included in the analysis between June 1, 2011, and April 30, 2017.
